# Crystal Structure of the *Bacillus subtilis* Phosphodiesterase PhoD Reveals an Iron and Calcium-containing Active Site^♦^

**DOI:** 10.1074/jbc.M114.604892

**Published:** 2014-09-12

**Authors:** Fernanda Rodriguez, James Lillington, Steven Johnson, Christiane R. Timmel, Susan M. Lea, Ben C. Berks

**Affiliations:** From the ‡ Department of Biochemistry, University of Oxford, South Parks Road, Oxford OX1 3QU,; the § Sir William Dunn School of Pathology, University of Oxford, South Parks Road, Oxford OX1 3RE, and; the ¶ Inorganic Chemistry Laboratory, University of Oxford, South Parks Road, Oxford OX1 3QR, United Kingdom.

**Keywords:** Bacterial Metabolism, Catalysis, Metalloenzyme, Phosphatase, Structural Biology

## Abstract

The PhoD family of extra-cytoplasmic phosphodiesterases are among the most commonly occurring bacterial phosphatases. The exemplars for this family are the PhoD protein of *Bacillus subtilis* and the phospholipase D of *Streptomyces chromofuscus.* We present the crystal structure of *B. subtilis* PhoD. PhoD is most closely related to purple acid phosphatases (PAPs) with both types of enzyme containing a tyrosinate-ligated Fe^3+^ ion. However, the PhoD active site diverges from that found in PAPs and uses two Ca^2+^ ions instead of the single extra Fe^2+^, Mn^2+^, or Zn^2+^ ion present in PAPs. The PhoD crystals contain a phosphate molecule that coordinates all three active site metal ions and that is proposed to represent a product complex. A C-terminal helix lies over the active site and controls access to the catalytic center. The structure of PhoD defines a new phosphatase active site architecture based on Fe^3+^ and Ca^2+^ ions.

## Teaser

**Background:** PhoD family enzymes liberate phosphate from organic compounds.

**Results:** The structure of PhoD reveals an active site with one Fe^3+^ and two Ca^2+^ ions.

**Conclusion:** PhoD represents a new class of phosphatase related to purple acid phosphatases.

**Significance:** The requirement of PhoD for iron ions may limit bacterial phosphate acquisition in low iron environments.

## Introduction

Under conditions of phosphate deficiency bacteria attempt to mobilize phosphate from organic molecules by synthesizing extra-cytoplasmic alkaline phosphatase enzymes. Analysis of the Global Ocean Survey metagenomic data set (1) suggests that these extra-cytoplasmic alkaline phosphatases come predominantly from the PhoA, PhoX, and PhoD families, with genes encoding PhoD enzymes being as abundant as those of the other two enzyme families combined (2).

PhoA family enzymes are predominantly phosphomonoesterases. The active site of the prototypical PhoA enzyme of *Escherichia coli* contains two Zn^2+^ and one Mg^2+^ ion, and the catalytic mechanism involves an enzyme-bound phosphoserine intermediate (3). PhoX enzymes are exclusively phosphomonoesterases, and their complex active site cofactor has recently been shown to contain two Fe^3+^ ions, three Ca^2+^ ions, and a μ_3_-bridging oxo group (4).

Enzymes of the PhoD family are the least well characterized of the three major classes of bacterial extra-cytoplasmic alkaline phosphatase. The archetype of the family is *Bacillus subtilis* PhoD. This enzyme is a phosphodiesterase but also has significant phosphomonoesterase activity (5). Metal ion reconstitution experiments show that PhoD requires Ca^2+^ for activity (5). The physiological role of PhoD is proposed to be the release of phosphate from cell wall teichoic acids under conditions of phosphate starvation (6). A second biochemically characterized member of the PhoD family is the secreted phospholipase D (PLD)^7^ from *Streptomyces chromofuscus* (7). PLD cleaves the phosphodiester bond that links the head group and diacyl glycerol portions of phospholipids. PLD is reported to contain one iron atom together with substoichiometric amounts of manganese. In addition, the enzyme requires Ca^2+^ ions for significant activity and to bind to phospholipid membranes.

Sequence analysis suggests that PhoD proteins are unrelated to the PhoA or PhoX phosphatase families but instead form a distinct subgroup within a very broad structural superfamily of hydrolytic enzymes possessing binuclear metal centers at the active site (the “metallophosphatase” superfamily in the Conserved Domain Database (8)). Within this structural superfamily it has been argued that the PhoD family has greatest similarity to eukaryotic purple acid phosphatases (PAPs) (9). In PAPs the active site metal pair are a Fe^3+^ ion and a divalent ion that can be Fe^2+^, Mn^2+^, or Zn^2+^ depending on the enzyme (10, 11). The Fe^3+^ ion is ligated by a tyrosine ligand resulting in an intense phenolate-to-Fe(III) charge-transfer transition (λ_max_ = 510–560 nm; ϵ = ∼3000–4000 m^−1^ cm^−1^), which gives PAPs their characteristic purple color. The occurrence of a tyrosinate-ligated Fe^3+^ ion is highly characteristic for PAPs, with the only other currently identified examples being found in the structurally unrelated diferric DNA ligase from *Ferroplasma acidiphilum* (12). It is, thus, striking that *S. chromofuscus* PLD exhibits a PAPS-like visible spectrum. Amino acid residues, including a tyrosine, that could correspond to the Fe^3+^ ligands in PLD have been proposed on the basis of sequence conservation and mutagenesis experiments (9).

In an effort to define the active site architecture of the PhoD phosphatase family we have determined the structure of *B. subtilis* PhoD. Unexpectedly, the active site contains three metal ions, these being identified as a Fe^3+^ ion and two Ca^2+^ ions. Our structural data show that the PhoD active site is distinct from that found in PAPs but that these two enzyme families conserve the position and coordination environment of their Fe^3+^ ions. A phosphate molecule bound to the PhoD active site gives insight into the mechanism of catalysis.

## EXPERIMENTAL PROCEDURES

### 

#### 

##### Cloning

The sequence coding for the mature domain of PhoD (codons 57–593) was amplified from *B. subtilis* strain 168 chromosomal DNA using the primers 5′-CCAGTGGGTCTCAGGTGGTGCGCCTAACTAAGC-3′ and 5′-CGGCGTCGACTTAATTCGTGATTTTTGCACG-3′. The resulting amplicon was digested with BsaI and SalI and cloned into the same sites in plasmid pCA597 (13) to produce plasmid pCA597-*mphoD*. This plasmid directs the expression of the mature domain of PhoD with a Ulp1-cleavable N-terminal Strep-SUMO tag (where SUMO is small ubiquitin-like modifier). Site-specific mutations were introduced into the *phoD* coding sequence in plasmid pCA597-*mphoD* using the QuikChange^TM^ method (Stratagene).

##### Protein Purification

*E. coli* strain BL21 (DE3)(14) containing plasmids pCA597-*mphoD* and pRARE (Novagen) was grown aerobically at 37 °C in 2×YT medium (Sigma-Aldrich) supplemented with 50 μg/ml kanamycin. Protein expression was induced at an *A*_600 nm_ = 0.6 with 0.5 mm isopropyl-1-thio-β-d-galactopyranoside, and the culture grown was then grown for 15 h at 20 °C.

Cells were harvested by centrifugation and resuspended in resuspension buffer (20 mm Hepes NaOH, pH 7.4, 200 mm NaCl, 1 mm CaCl_2_), with EDTA-free complete protease inhibitors (Roche Applied Science) and DNase I (Sigma-Aldrich). The cells were broken by three passages through a French press at 8000 p.s.i. The lysate was clarified by successive centrifugation at 10,000 × *g* for 20 min at 4 °C and at 45,000 × *g* for 1 h at 4 °C, and then applied to a 5-ml self-packaged Strep-Tactin column (IBA, GmbH, Göttingen, Germany). Recombinant PhoD was eluted from the column with resuspension buffer containing 2.5 mm desthiobiotin and then supplemented with 1 mg of His_6_-Ulp1 protease (13) and dialyzed overnight at 4 °C against resuspension buffer. Cleaved tags and uncleaved mPhoD protein were removed by incubation with Strep-Tactin matrix, and the sample was further purified on a Superdex75 (16/60) prep grade size-exclusion column (GE Healthcare) that had been equilibrated in resuspension buffer. The mPhoD-containing fractions were pooled and concentrated using a 30-kDa cut-off Amicon PLTK Ultracel-PL membrane concentrator (Merck Millipore).

##### Phosphatase Activity Assays

Alkaline phosphatase activity was assayed spectrophotometrically by measuring the release of *p*-nitrophenol from *p*-nitrophenyl phosphate (pNPP) or bis-*p*-nitrophenyl phosphate (bis-pNPP). Assays were started by the addition of 1 μg/ml PhoD to 100 mm Tris-HCl, pH 8.0, 2 mm CaCl_2_, and 1 mm pNPP or bis-pNPP at 25 °C. Reactions were stopped after 5 min by the addition of 1 m final concentration NaOH. *p*-Nitrophenol production was quantified using ϵ_405 nm_ = 18,000 m^−1^ cm^−1^.

The substrate specificity of PhoD was assessed by measuring phosphate release from test substrates. Each 800-μl reaction contained 100 mm Tris-HCl, pH 8.0, 2 mm CaCl_2_, 1 mm test substrate, and 1 μg of PhoD. After a 5-min incubation at 25 °C the reaction was terminated by the addition of 200 μl of malachite green reagent (AMS Biotechnology) followed by incubation at room temperature for 30 min. Production of phosphate was obtained from absorbance at 620 nm and quantified using a phosphate standard curve.

##### Crystallographic Data Collection and Structure Determination

Crystallization trials were performed at 294 K by the sitting drop vapor diffusion method. The best crystals were obtained in 0.2 m NaCl, 0.1 m Na_2_HPO4/KH_2_PO4, 54% PEG 200, pH 5.6, with protein at 7.8 mg/ml, a total drop volume of 400 nl, and a 50:50 protein-to-mother liquor ratio. The crystals were cryo-protected in mother liquor containing 10% (w/v) ethylene glycol and flash-cooled in liquid nitrogen. X-ray data were collected at beamlines ID23_1 (European Synchrotron Radiation Facility (ESRF), Grenoble, France) and I03 (Diamond Light Source, Oxfordshire, UK). The data were processed in space group I4122 using Process (part of the autoPROC pipeline; Global Phasing Ltd.) (15). The multiple wavelength anomalous diffraction method was used to determine the sites of the three catalytic metals (initially assigned as three iron sites, but later refined as one iron and two calcium sites) using autoSharp (16) and to produce phases that were optimally solvent-flattened to 62% using Solomon (17). The handedness of the sites was determined by the contrast (standard deviation of solvent to standard deviation of protein) falling from 57 to 27% in one hand and from 63 to 39% in the other. Buccaneer (18) built a partial model among a number of other unlikely chains. This model was then manually manipulated in accordance with the kidney bean purple acid phosphatase structure (Protein Data Bank (PDB) 3KBP). Input of this partial model for a second round of Buccaneer building placed 524 residues of a monomer into the map. This was used as the search model for molecular replacement via MOLREP (19), placing the two translational related non-crystallographic symmetry copies at (0.5, 0.5, 0.08) of the unit cell from one another. Iterative refinement and model building were carried out with Refmac (20) and Coot (21). The quality of the model was checked using MolProbity (22), with a final MolProbity score of 1.19 (93th percentile).

The sample for proton-induced x-ray emission (micro-PIXE) analysis was purified in a calcium-free buffer composed of 20 mm Tris-HCl, pH 7.2, 200 mm potassium acetate. Measurements were taken at the Ion Beam Centre (University of Surrey).

##### EPR Spectroscopy

EPR spectra were collected on an X-band CW EPR spectrometer (Bruker Biospin GmbH) using an X-band Super High Sensitivity Probehead (Bruker) and equipped with a low temperature helium flow cryostat (Oxford Instruments CF935). Data were analyzed using the program EasySpin (23).

## RESULTS

### 

#### 

##### Characterization of B. subtilis PhoD

**FIGURE 1.**
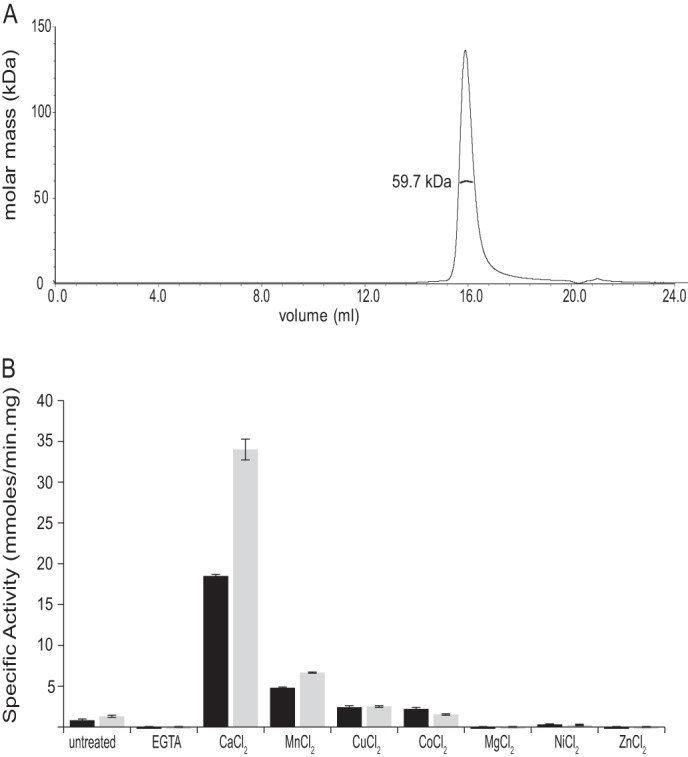
*A*, size-exclusion multi-angle laser light scattering analysis of PhoD. The buffer was 20 mm Hepes, pH 7.5, 400 mm NaCl, 2 mm calcium acetate, and the flow rate was 0.4 ml/min. A single species of weight-averaged molecular mass 59,700 Da (± 1%) is observed. *B*, phosphomonoesterase and phosphodiesterase activities of PhoD measured with pNPP (*black bars*) and bis-pNPP (*gray bars*), respectively. PhoD in 5 mm Tris-HCl, pH 8.0, was treated with 100 μm Na_2_EGTA for 5 min (bar marked *EGTA*), and then 2 mm of the chloride salt of the indicated divalent metal cation was added. *Error bars* indicate the S.D. of three independent experiments.

**FIGURE 2.**
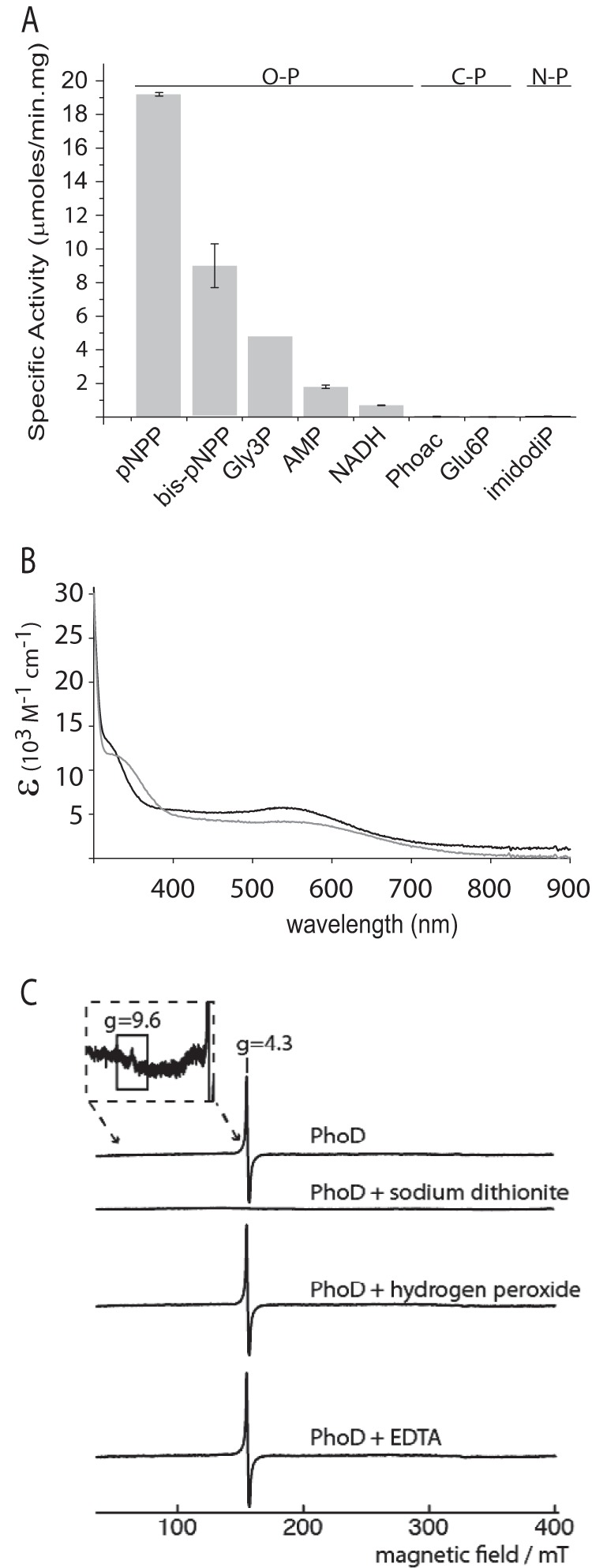
**Characterization of recombinant *B. subtilis* PhoD.** *A*, phosphate released from different substrates by PhoD. The assays contained 1 μg/ml PhoD and 1 mm of the test substrate in 100 mm Tris, pH 8.0, 2 mm CaCl_2_. *Gly3P* is glycerol 3-phosphate; *Phoac* is phosphonoacetic acid; *Glu6P* is glucose 6-phosphate; *imidodiP* is imidodiphosphate. Substrates are grouped according to whether the target bond is between phosphorus and oxygen (O–P), phosphorus and carbon (C–P), or phosphorus and nitrogen (N–P). *Error bars* indicate the S.D. of three independent experiments. *B*, visible absorption spectrum of 2.3 mg/ml PhoD in 20 mm Hepes NaOH, pH 7.4, 200 mm NaCl, 1 mm CaCl_2_ in the absence (*black*) or presence (gray) of 10 mm Na_2_EGTA. *C*, EPR spectra of PhoD highlighting the g = 4.3 peak. Spectra are shown for native PhoD, PhoD with 5 mm sodium dithionite, PhoD with 2 mm hydrogen peroxide, and PhoD with 50 mm EDTA. The *inset* shows a magnified part of the spectrum around g = 9.6. The PhoD concentration was 10 mg/ml in 20 mm Hepes, pH 7.5, 400 mm NaCl, 2 mm calcium acetate with 20% (v/v) glycerol added. A control experiment showed that the glycerol added to the samples did not change the spectral line shape (data not shown). EPR operating conditions: microwave power, 2 milliwatts; microwave frequency, 9.425 GHz; modulation, 5.0 G at 100 kHz; temperature, 10 K. Spectra are buffer-subtracted, with negative controls taken for sodium dithionite, hydrogen peroxide, and EDTA solutions.

*B. subtilis* PhoD was heterologously expressed in the cytoplasm of *E. coli*. Purified recombinant PhoD protein was a monomer in solution as judged by size-exclusion multi-angle laser light scattering (Fig. 1*A*). The recombinant PhoD exhibited phosphomonoesterase activity with the colorigenic substrate pNPP and exhibited phosphodiesterase activity with the colorigenic substrate bis-pNPP (Fig. 1*B*). However, the specific activity of the recombinant enzyme was very much lower than previously reported for the native enzyme (5). We observed that the activity of the recombinant enzyme was massively enhanced by Ca^2+^ supplementation (Fig. 1*B*), and so Ca^2+^ was subsequently included in all buffers used for the purification and analysis of PhoD. At pH 8.0 and a temperature of 25 °C, the Ca^2+^-supplemented recombinant PhoD protein had a *K_m_* for pNPP of 50 μm and a *k*_cat_ of 1.2 s^−1^. PhoD was able to release phosphate from compounds containing phosphoester bonds but was not able to cleave phosphorus-nitrogen or phosphorus-carbon bonds (Fig. 2*A*).

PhoD purified in the presence of Ca^2+^ was purple in color with a broad absorbance band in the visible spectrum, indicating the presence of a prosthetic group (Fig. 2*B*). The addition of the Ca^2+^-selective chelator EGTA caused a shift in the visible absorbance maximum from around 540 nm to ∼560 nm (Fig. 2*B*), suggesting that Ca^2+^ is associated with the prosthetic group. Proton-induced x-ray emission spectroscopy of PhoD purified in the absence of added Ca^2+^ ions detected iron and calcium but no cobalt, magnesium, manganese, nickel, or zinc. The presence of iron in PhoD was confirmed by EPR spectroscopy, which detected the g = 4.3 and weak g = 9.6 peaks characteristic of the middle and lower Kramer's doublets of an isolated high spin rhombic Fe^3+^ ion (Fig. 2*C*). No further signals were elicited by oxidation with hydrogen peroxide. All EPR signals disappeared on reduction with sodium dithionite, as expected of reduction of a monomeric Fe^3+^ ion to the EPR silent Fe^2+^ state (Fig. 2*C*). The Fe^3+^ signal was unperturbed by the addition of 50 mm EDTA (Fig. 2*C*), showing that the iron in PhoD is too tightly bound to be removed by chelator treatment. The presence of iron in PhoD has not previously been reported. However, iron is found in the homologous *S. chromofuscus* PLD enzyme (7).

##### Structure of PhoD

PhoD was crystallized at pH 5.6 in the presence of phosphate. The structure of PhoD was determined to a resolution of 2 Å via a multi-wavelength anomalous diffraction experiment using the iron cofactor as the source of the anomalous signal. Final *R*/*R*_free_ values at this resolution were 17.8/20.2%. Data reduction, phasing, and crystallographic refinement statistics are shown in Table 1. Every residue in the PhoD mature domain was observed in the electron density.

**TABLE 1:**
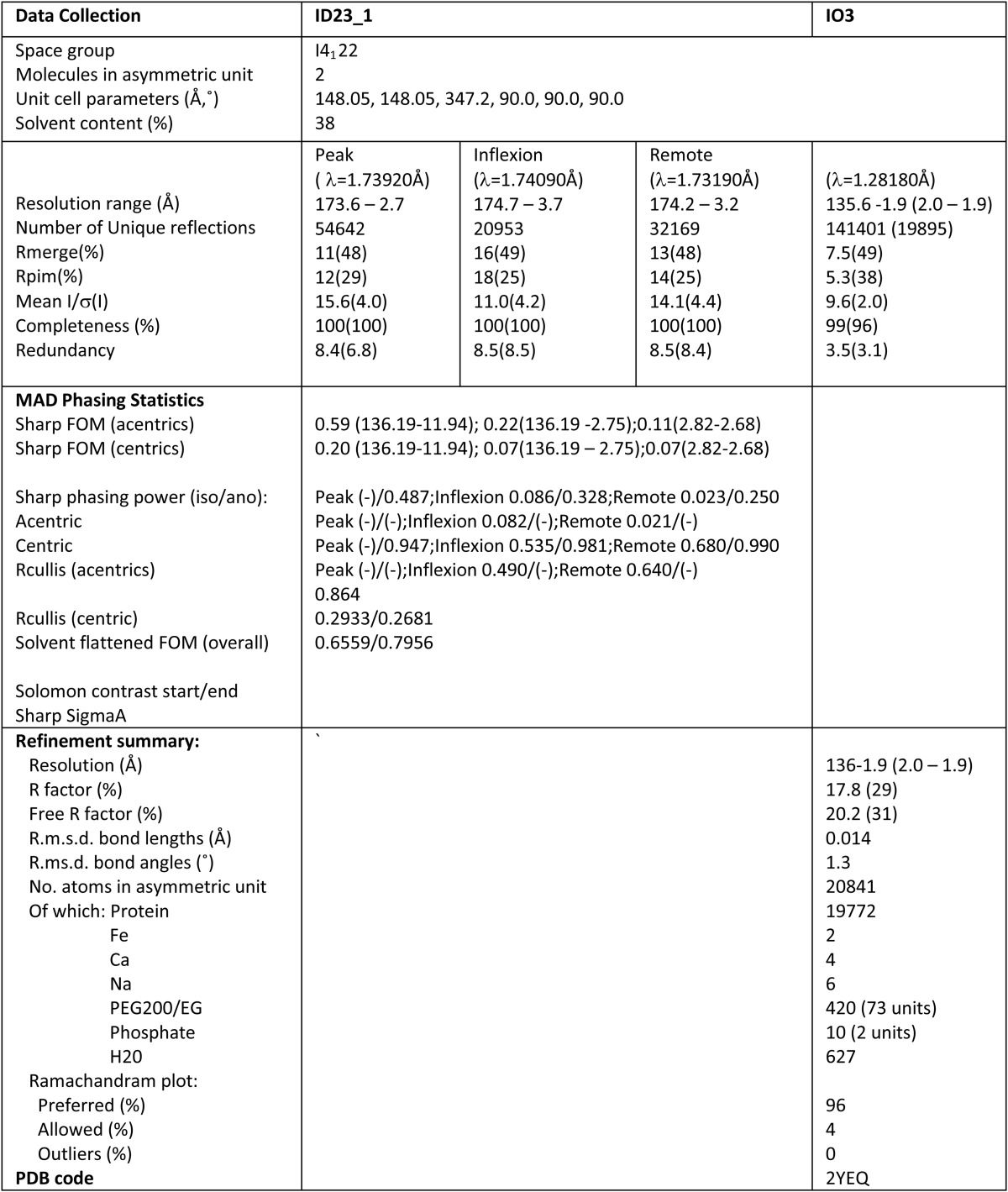
**Data collection and refinement statistics**

**FIGURE 3.**
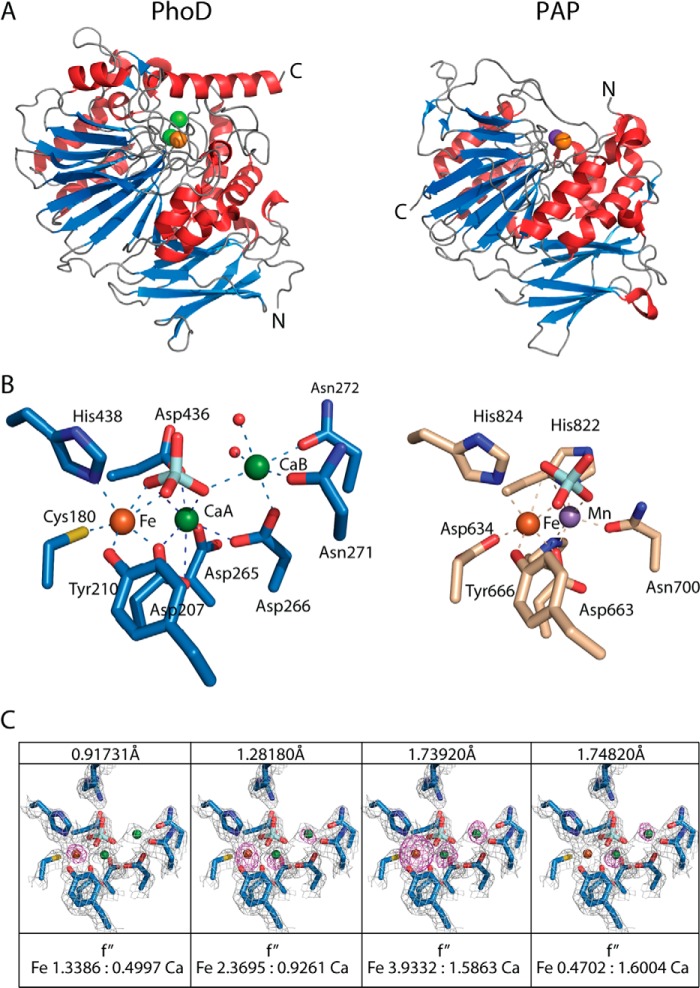
**PhoD has structural similarity to purple acid phosphatases.** *A*, comparison of the overall fold of PhoD (*left*) and sweet potato PAP (PDB 1XZW) (*right*). Both structures are shown in the same orientation with β-strands colored *blue* and α-helices colored *red*. The catalytic site metal ions are presented as *spheres*, with Fe^3+^ shown in *orange*, Ca^2+^ shown in *green*, and Mn^2+^ shown in purple. *B*, comparison of the coordination environment of the PhoD metal ions (*left*) with those in sweet potato PAP (*right*). Metal ions are colored as in *A*, and other atoms are colored as follows: oxygen in *red*; nitrogen in *dark blue*; sulfur in *yellow*; phosphorus in *light blue*; PhoD carbons in *sky blue*; and PAP carbons in *tan*. Metal ion coordination bonds are shown by *dashed lines*. Alignment between the proteins was carried out with LSQKAB (44). *C*, electron density at the PhoD active site contoured at 1σ (*gray*) overlaid with anomalous Fourier maps contoured at 4σ (*purple*) at the x-ray wavelengths indicated at the top of each panel. The anomalous signals for iron relative to calcium are consistent with the theoretical f″ values for iron and calcium at these wavelengths calculated using Crossec (45), which are given under the panels.

**FIGURE 4.**
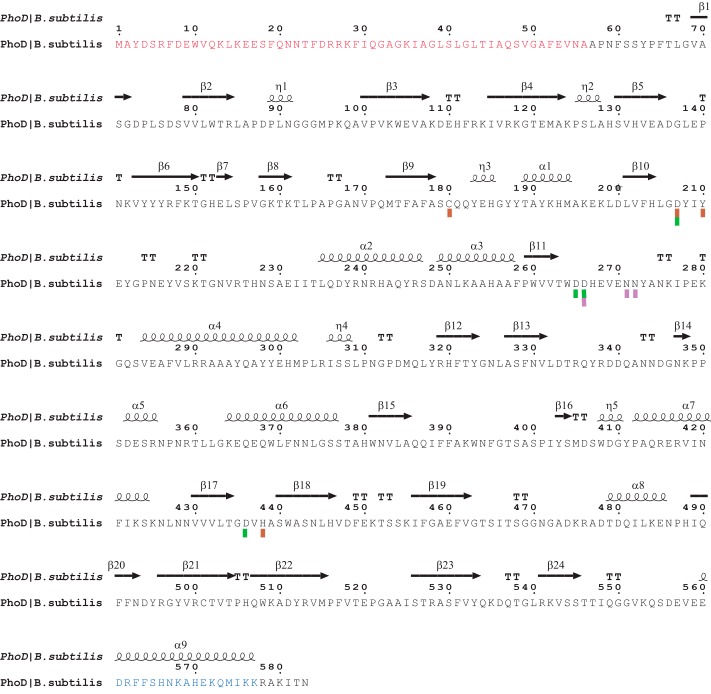
**Amino acid sequence of PhoD.** The secondary structure elements are shown above the sequence, with α-helices represented by *squiggles*, β strands represented by *arrows*, and turns represented by the letters *TT*. The signal peptide is highlighted in *red*, and the C-terminal α-helix is highlighted in *blue*. Metal ligating residues are indicated by *blocks* beneath the sequence, with those coordinating the Fe ion in *brown*, Ca_A_ in *green*, and Ca_B_ in *purple*. Residues that coordinate two metal ions are indicated by *blocks with two colors*.

**FIGURE 5.**
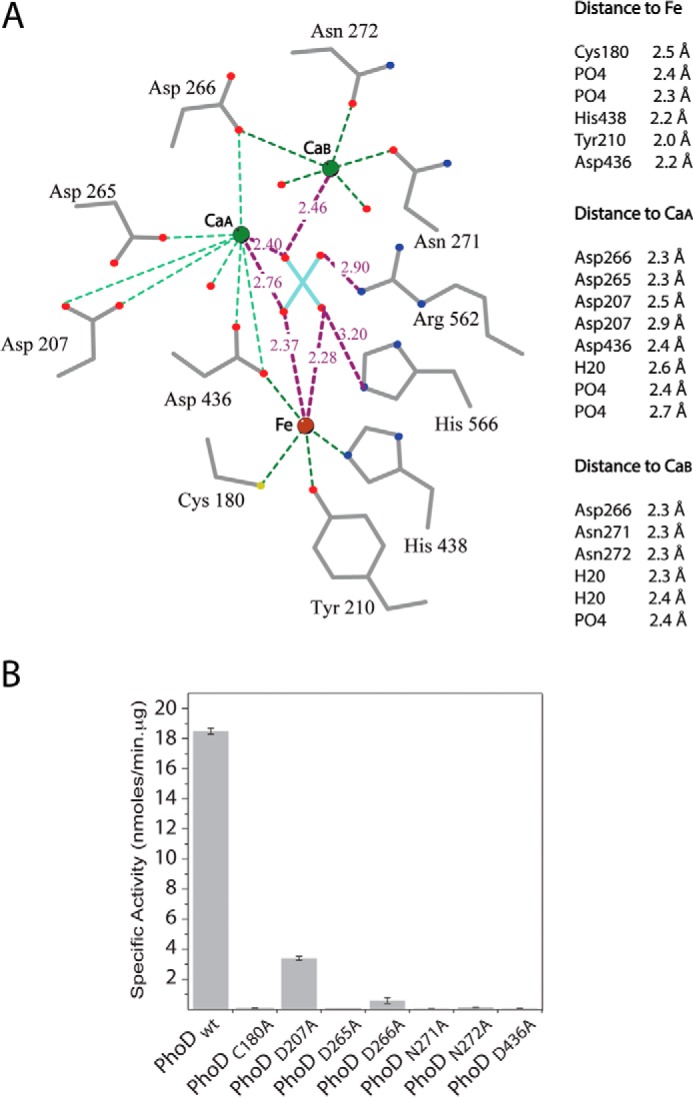
**Characterization of the PhoD active site.** *A*, diagrammatic representation of bonding interactions in the PhoD active site. Amino acid side chains and the bound phosphate ion are shown in sticks representation with carbon atoms in *gray*, oxygen atoms in *red*, nitrogen atoms in *blue*, phosphorus atom in *cyan*, and sulfur atom in *yellow*. Bonding interactions are represented by *dotted lines*. Bond distances in Å to the phosphate ion are marked on the figure. Other metal ion-ligand distances are tabulated at the *right-hand side* of the figure. The figure was drawn using LIGPLOT (46). *B*, phosphatase activity of PhoD active site variants using pNPP as substrate. PhoD wt is the wild-type protein. *Error bars* indicate the S.D. of three independent experiments.

The core of the PhoD structure is a sandwich of two long β-sheets flanked by α-helices of varying lengths (Figs. 3*A* and 4). Three active site metal ions are coordinated by residues on loops on one face of the β-sandwich and are covered by a long C-terminal helix. With the knowledge that PhoD contains Fe^3+^ and Ca^2+^ ions (above) anomalous Fourier maps at three wavelengths allowed the assignment of the three active site metal ions as one Fe^3+^ ion and two Ca^2+^ ions, which we label Ca_A_ and Ca_B_ (Figs. 3, *B* and *C*, and 5*A*). Substitution of individual amino acids involved in metal ion coordination at each of the three metal sites either abolished (C180A, D265A, N271A, N272A, and D436A variants) or drastically reduced (D207A and D266A variants) PhoD activity with pNPP as a substrate (Fig. 5*B*), showing that all three metal ions are required for the phosphomonoesterase activity of PhoD.

PhoD was crystallized in the presence of phosphate, and the structure contains a phosphate molecule bound at the active site (Fig. 3*B*). Phosphate is a known competitive inhibitor of PhoD (5).

##### PhoD Has Structural Similarity to Purple Acid Phosphatases

A search of the PDB protein structure database using the DALI server (24) revealed that PhoD has highest structural similarity to PAPs. The closest overall structural match was to red kidney bean PAP (PDB 4DHL) (25) with a DALI *Z*-score of 28.6 and a main chain root mean square deviation relative to PhoD of 2.47 Å. The similarity between the two protein families is illustrated in Fig. 3, *A* and *B*, where we have chosen to compare PhoD with sweet potato PAP (PDB 1XZW) (26) due to the close structural homology in the way the two proteins bind phosphate (discussed below).

At the Fe^3+^ site the sole difference in amino acid ligand sets between PhoD and the PAPs is a cysteine-for-aspartate substitution in PhoD (Cys-180). Cysteine is an unusual ligand to a Fe^3+^ ion. Indeed, to the best of our knowledge, the structurally unrelated alkaline phosphatase PhoX (4) is the only other protein using a thiolate group to coordinate a redox-inactive Fe^3+^ ion. The visible absorbance bands of PhoD are likely to arise predominantly from the tyrosine ligation of the Fe^3+^ ion, as in the PAPs (27). However, additional thiolate-to-Fe^3+^ charge transfer bands from the cysteine ligand will also contribute to the visible spectrum (28).

The Ca_A_ site in PhoD corresponds to the position of the second metal site in the PAPs. Nevertheless, there is little similarity between the metal ion ligands found at this site in the two types of protein (Fig. 3*B*), consistent with the different coordination preferences of Ca^2+^ and the divalent metal ions found in the PAPs. Specifically, the Ca_A_ site has irregular 8-fold coordination with all amino acid ligation being provide by Asp residues.

The Ca_B_ site in PhoD has no analogue in the PAPs. The Ca_B_ ion has regular octahedral ligation with Asp and Asn ligands.

**FIGURE 6.**
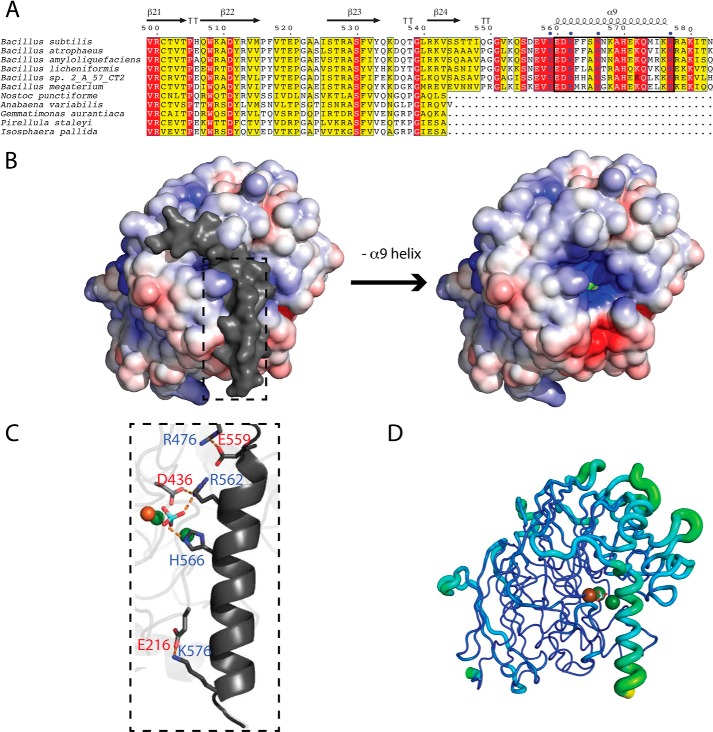
**The C-terminal helix of PhoD occludes the active site.** *A*, multiple sequence alignment of the C termini of PhoD family proteins showing the extension seen in the PhoD protein of *B. subtilis* and some other *Bacillus* species. Secondary structure elements from the *B. subtilis* PhoD structure are shown above the alignment with the α-helix shown as a *coil*, β-strands shown as *arrows*, and β-turns shown by the *letter T*. Conserved amino acids are highlighted in *red*, and similar amino acids are highlighted in *yello*w. The *black box* indicates the C-terminal α-helix of *B. subtilis* PhoD. Residues in the C terminus involved in salt bridges with the main structure (colored *blue*) are indicated with *blue dots* above the alignment. *B*, electrostatic surface model (± 5 *kT*/*e*) of intact PhoD (*left*) and with the C-terminal helix removed (*right*). Regions of negative charge are shown in *red*, and regions of positive charge are shown in *blue*, but with the C-terminal helix in *gray*. Electrostatic calculations were performed with APBS (47) and include the charges on the active site metal ions. *C*, the salt bridge network between the C-terminal helix, the bound phosphate ion, and the active site. The portion of PhoD shown corresponds to the region of the PhoD structure highlighted in *panel B. D*, B-factor putty diagram of PhoD generated by PyMOL. Flexibility is indicated by increasing ribbon girth and color scaling from *blue* to *green*.

All three metal ions in PhoD have bonding interactions with the phosphate group present in the active site (Figs. 3*B* and 5*A*). One oxygen atom of the phosphate ligates the iron, one oxygen atom bridges Ca_A_ and the iron, and one oxygen atom bridges the two calcium ions. The interactions between the phosphate and the Ca_A_-Fe pair replicate the tripodal μ–η2–η2-geometry observed between phosphate and the two metal ions of sweet potato PAP in PDB structure 1XZW (26)(Fig. 3*B*). A similar mode of bridging tripodal coordination has also been seen for phosphate bound to the dimetal centers of *Sporosarcina pasteurii* urease (29) and an organophosphate-degrading enzyme from *Agrobacterium radiobacter* (30). In addition to interacting with the active site metal ions the phosphate ion in PhoD has bonding interactions with the amino acid side chains of Arg-562 and His-566 from the C-terminal capping helix (Figs. 5*A* and 6, *A–C*).

##### The C-terminal Helix Occludes Access to the PhoD Active Site

The C-terminal region of the *B. subtilis* PhoD protein forms an α-helix (residues 560–577), which lies over the active site (Figs. 3*A* and 6). This helix is part of a C-terminal extension that is found in PhoD from *B. subtilis* and certain additional *Bacillus* species, but is absent from other bacterial PhoD proteins and from the PAPs (Fig. 6*A*). The C-terminal helix is held in position by a network of salt bridges, which include interactions with the phosphate ion located in the active site, and with Asp-436, which bridges the Fe and Ca_A_ ions (Fig. 6*C*).

The C-terminal helix blocks access to the catalytic site. Furthermore, as judged by modeling studies with pNPP substituted for the phosphate molecule seen in the crystal structure, steric clashes with the helix would also prevent substrates binding to the active site. As a consequence, the C-terminal helix must move from the position seen in the crystal structure in order for substrates to bind to PhoD. The main chain crystallographic B-factor for the C-terminal helix is high (49.1 Å^2^) relative to the whole protein average (28.2 Å^2^), showing that the C-terminal helix is more mobile than the rest of the PhoD structure (Fig. 6*D*). This suggests that the helix is relatively weakly bound to the main body of PhoD and could plausibly be released at some stage of the catalytic cycle. Attempts to observe the predicted movement of the C-terminal helix by co-crystallization with the bulky substrate analogues phosphonoacetic acid and phosphonohexanoic in place of phosphate were unsuccessful.

**FIGURE 7.**
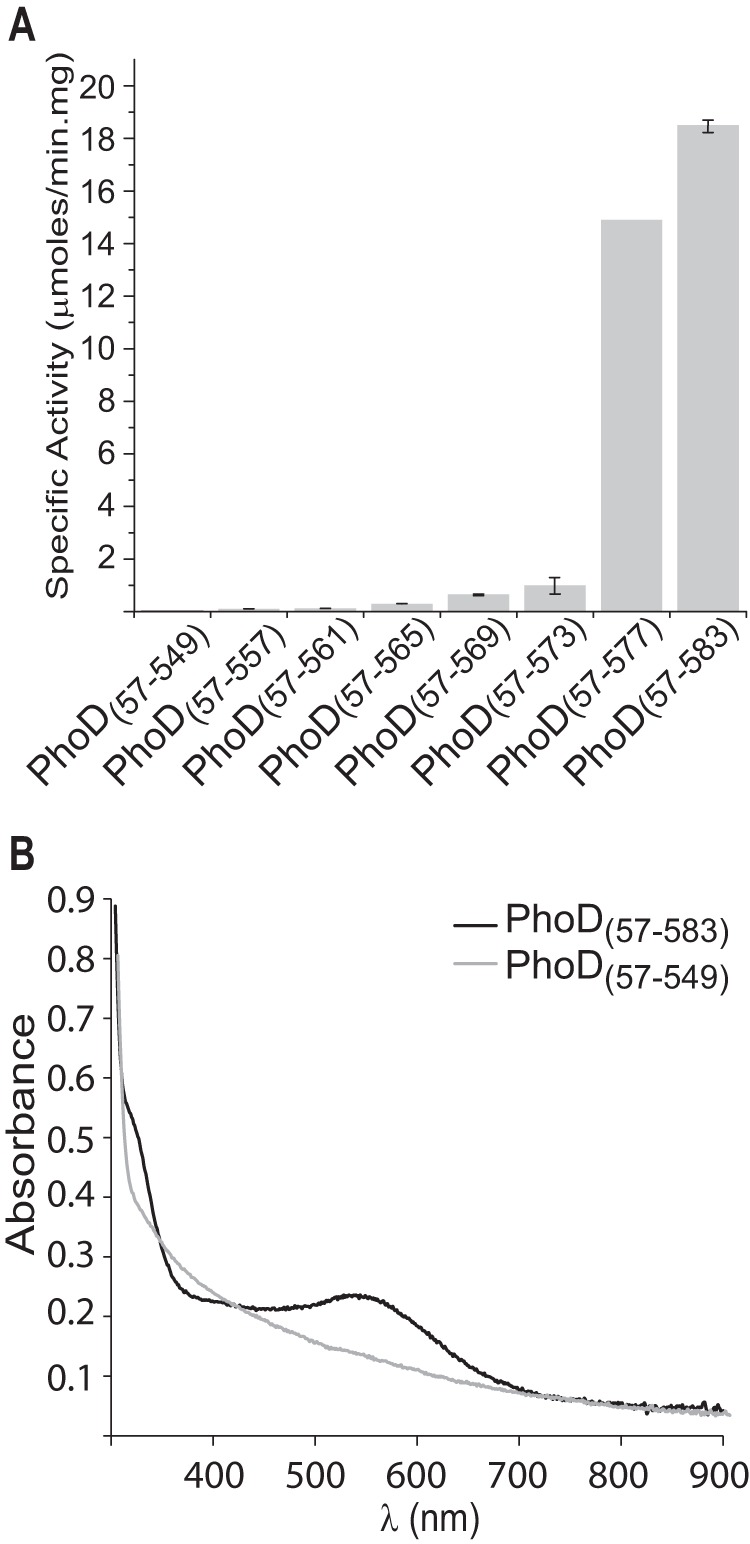
**The C-terminal helix of PhoD is functionally important.** *A*, phosphatase activity of C-terminally truncated PhoD variants. PhoD (57–583) is the intact protein. Activities were measured using pNPP as substrate. *Error bars* indicate the S.D. of three independent experiments. *B*, visible absorption spectra of wild-type PhoD (57–583)(*black line*) and the C-terminal truncation variant (57–549)(*gray line*).

The function of the PhoD C-terminal helix was investigated using variants possessing truncations of this helix. These experiments show that the whole C-terminal helix is required for catalytic activity and that even the variant with the smallest truncation tested had lost the visible absorbance bands arising from the Fe^3+^ ion (Fig. 7). Thus the C-terminal helix is required to either insert or retain the PhoD active site metal cofactor.

## DISCUSSION

The structure of *B. subtilis* PhoD confirms earlier suggestions (9) that PhoD family proteins have structural features in common with eukaryotic PAPs. The two protein families share the same protein fold, and both contain a tyrosinate-ligated Fe^3+^ ion at the same position in the active site. Indeed, the sole difference in the Fe^3+^ coordination environment between PAPS and *B. subtilis* PhoD is the substitution of one of the aspartic acid residues in the canonical PAPs ligand set by a cysteine (Cys-180). In this context it is worth noting that PhoD family proteins can be divided on sequence grounds into two subfamilies, one of which possesses the cysteine Fe^3+^ ligand seen in *B. subtilis* PhoD and also *S. chromofuscus* PLD, whereas the second has the aspartate ligand found in the PAPS site.

Notwithstanding their similarities, the PhoD and PAPs families clearly form distinct sequence groups and differ in their active site metal complement beyond the tyrosine-ligated Fe^3+^ ion. Although the PAPs bind an additional Fe^2+^, Mn^2+^, or Zn^2+^ ion, *B. subtilis* PhoD binds two Ca^2+^ ions. Other members of the PhoD family almost certainly bind the same two Ca^2+^ ions because the coordinating amino acids are close to invariant within the family (the residue equivalent to *B. subtilis* PhoD Ca_B_ ligand Asn-272 is sometimes conservatively substituted with an Asp, which is also a good Ca^2+^ ligand). Although the position of the Ca_A_ ion in PhoD is similar to that of the divalent ion in the PAPs, the ligand sets provided by the two protein families are completely distinct. Ca_A_ in PhoD has five coordination bonds from Asp residues, whereas the divalent ion in PAPs is ligated by an invariant His_2_Asp_1_Asn_1_ set (31). It should be noted that the earlier predictions for the non-iron ion ligands in the PhoD family are incorrect (9). A few bacteria encode proteins that conserve all PAP metal ligands (32), and these proteins can be considered to be true bacterial PAPs. By contrast, our structural analysis shows that the much more widely distributed enzymes of the PhoD family should be regarded as forming a distinct but closely related sister group to the PAPs. Because *B. subtilis* PhoD and *S. chromofuscus* PLD are thought to target specific phosphate-containing molecules *in vivo* (teichoic acids and phospholipids, respectively) it is possible that other PhoD family members also have specialist biological roles rather than operating as general phosphatases like members of the PhoA and PhoX families.

*B. subtilis* PhoD has a flexible C-terminal helix overlaying the active site (Fig. 7). Although this helix is not present in most other members of the PhoD family, analogous “cap domains” are a feature of certain other enzymes in the metallophosphatase superfamily (33–36). In most cases the role of these cap domains is unclear. Intriguingly, however, the cap domain of a mycobacterial metallophosphatase has recently been implicated in cell wall binding (33). An equivalent function for the PhoD C-terminal helix is an attractive hypothesis given the proposed role of PhoD in teichoic acid metabolism.

The way in which phosphate is bound to the active site of PhoD in our crystal structure is likely to mimic the substrate-enzyme interaction at some point in the catalytic reaction. We can get considerable insight into the likely mechanism of PhoD catalysis by interpreting the phosphate complex in the light of the extensive body of work on the mechanism of PAPs. In PAPs the two active site metal ions are bridged by a hydroxide ion. The current view is that phosphomonoester substrates coordinate both PAP metal ions. The bridging hydroxide ion then undertakes a nucleophilic attack on the phosphorus atom in-line with the bond to the leaving group. This results in the hydroxide ion being incorporated into the phosphate product (10). A structure has been determined for sweet potato PAP in which a phosphate ion is bound in a tripodal μ–η2–η2-geometry between the two metals ions such that one of the oxygen atoms occupies the position normally taken by the bridging hydroxide ion (26). This structure has been interpreted as representing the initial product complex in which the oxygen atom of the hydroxide nucleophile is fully bonded to the substrate phosphorus atom while still maintaining bridging coordination to the metal ions. The position of the phosphate ion in the PhoD structure is highly analogous to that in the sweet potato PAPs structure (Fig. 3*B*), suggesting that PhoD possesses a nucleophilic hydroxide between the Fe and Ca_A_ ions and that the PhoD structure represents the initial product complex formed by the reaction involving this ion.

PhoD is a phosphodiesterase and so must be capable of catalyzing two successive hydrolysis reactions. This process could involve repeating the reaction involving the bridging hydroxide, with the initial product rotating in the active site before the second reaction. Alternatively, the two reactions could utilize distinct nucleophiles. Comparisons with the PAPs are again potentially informative in distinguishing between these two possibilities. Although PAPs have historically been considered not to catalyze phosphodiesterase reactions due to their inability to hydrolyze bis-pNPP (37) recent work with less sterically restricted substrates has shown that PAPs do possess this capability (38). The inferred phosphodiesterase mechanism in the PAPs is nucleophilic attack by a Fe^3+^-coordinated terminal hydroxide group followed by a second nucleophilic attack by the bridging hydroxide. A similar mechanism is plausible for PhoD with the phosphate complex corresponding to the initial product of the second hydrolytic reaction.

A striking difference between the PhoD and PAP families (and, indeed, other members of the metallophosphatase superfamily) is the presence of a third metal ion (Ca_B_) in PhoD. Because PAPs are able to catalyze phosphodiesterase reactions at a similar rate to phosphomonoester hydrolysis with only two metal ions (38) it is unlikely that the presence of CaB is specifically linked to the phosphodiesterase functionality of PhoD. Instead, Ca_B_ is appropriately placed to stabilize the developing charge on the leaving group during nucleophilic attack by the bridging hydroxide ion. More generally, the additional metal ion may compensate for the weaker polarizing ability of Ca^2+^ relative to the divalent metal ions found in the PAPs (Fe^2+^, Mn^2+^, Zn^2+^).

The combination of Fe^3+^ and Ca^2+^ ions used by PhoD is unusual and to the best of our knowledge has only otherwise been seen in the structurally unrelated PhoX alkaline phosphatase (4). Remarkably, therefore, two out of the three major classes of alkaline phosphatases in bacteria use the same, rare combination of metal ions. Both PhoD and PhoX are extra-cytoplasmic bacterial enzymes, and so the selection of Ca^2+^ as the divalent cation may simply reflect the high availability of Ca^2+^ ions in most terrestrial and marine environments (39). By contrast, the bioavailability of iron is low in many environments (40) and may limit the activity of PhoD family enzymes in such locations, as has also recently been argued for PhoX (4). Export of both PhoD and PhoX family proteins uses the Tat protein translocation system, which is dedicated to the transport of folded proteins (41–43). The Tat pathway is often used by proteins that need to fold before export to bind cofactor molecules in the cytoplasm. We speculate that the limited availability of soluble iron in the external environment means that biosynthesis of PhoD and PhoX cannot rely on the acquisition of adventitious iron in the periplasm. Iron ions would, instead, be inserted into these proteins under controlled conditions in the cytoplasm with export of the folded, iron-containing, proteins taking place via the Tat apparatus. In summary, the PhoD structure reveals that members of this widely distributed family of bacterial enzymes represent a new class of Fe^3+^- and Ca^2+^-dependent phosphatase.

## Footnotes

7 The abbreviations used are:

PLD: phospholipase D
PAP: purple acid phosphatase
pNPP: *p*-nitrophenyl phosphate
bis-pNPP: bis-*p*-nitrophenyl phosphate.
